# Impact of Concurrent Exercise Training on Cardiac Autonomic Modulation, Metabolic Profile, Body Composition, Cardiorespiratory Fitness, and Quality of Life in Type 2 Diabetes with Cardiac Autonomic Neuropathy: A Randomized Controlled Trial

**DOI:** 10.3390/jcm13133910

**Published:** 2024-07-03

**Authors:** Saima Zaki, Md Farhan Alam, Saurabh Sharma, Said El-Ashker, Mohammad Ahsan, Shibili Nuhmani

**Affiliations:** 1 Centre for Physiotherapy and Rehabilitation Sciences, Jamia Millia Islamia, Maulana Muhammad Ali Jauhar Marg, New Delhi 110025, India; saimazaki@jmi.ac.in (S.Z.); farhanphysio@gmail.com (M.F.A.); 2 Self-Development Department, Deanship of Preparatory Year, Imam Abdulrahman Bin Faisal University, P.O. Box 1982, Dammam 31441, Saudi Arabia; sgelashker@iau.edu.sa; 3 Department of Physical Therapy, College of Applied Medical Sciences, Imam Abdulrahman Bin Faisal University, Dammam 31441, Saudi Arabia; mahsan@iau.edu.sa (M.A.); snuhmani@iau.edu.sa (S.N.)

**Keywords:** glycemic control, metabolic control, autonomic dysfunction, cardiovascular disease, diabetes complications

## Abstract

**Background:** Type 2 diabetes mellitus (T2DM) often leads to cardiac autonomic neuropathy (CAN), a severe complication affecting cardiovascular health. Exercise training is a proven intervention for improving metabolic control and cardiovascular health in T2DM, but the effects of concurrent exercise training (CET), combining aerobic and resistance exercises, on CAN are not fully understood. **Objective:** This randomized controlled trial investigates the impact of a structured CET program on cardiac autonomic modulation, metabolic profile, body composition, cardiorespiratory fitness (CRF), and quality of life (QoL) in individuals with T2DM and CAN. **Methods:** A total of 96 participants, aged 35–70 years, with T2DM and CAN, were randomized into CET (*n* = 48) and control (*n* = 48) groups. The CET group engaged in combined aerobic and resistance training three times per week for 13 weeks, while the control group received standard care. Primary outcomes included heart rate variability (HRV) and heart rate recovery (HRR). Secondary outcomes were metabolic profile, body composition, CRF, and QoL, which were assessed using standardized protocols and validated questionnaires. The trial was registered with the Clinical Trials Registry—India (CTRI/2021/09/036711). **Results:** Significant improvements were noted in the CET group compared to controls. HRV metrics (SDNN, RMSSD, pNN50, TP, LF power, HF power, and LF/HF ratio) and HRR metrics (HRR30s, HRR1, HRR2, and HRR3) all showed significant enhancements (*p* < 0.01). The CET group also exhibited substantial reductions in fasting blood glucose, postprandial blood glucose, HbA1c, waist circumference, hip circumference, and percentage body fat (*p* < 0.01). Improvements were observed in lipid profile markers and CRF (VO_2max_) (*p* < 0.01). QoL scores improved significantly in the CET group as per the ADDQoL-19 (*p* < 0.01). **Conclusions:** CET significantly enhances cardiac autonomic modulation, metabolic profile, body composition, CRF, and QoL in individuals with T2DM and CAN. These findings support the integration of CET into standard T2DM management to improve clinical outcomes and QoL. Further research is needed to explore the long-term benefits and broader applicability of CET in diverse diabetic populations.

## 1. Introduction

Type 2 diabetes mellitus (T2DM) is a chronic metabolic disorder characterized by insulin resistance and hyperglycemia, leading to various complications, including cardiovascular disease and neuropathy [1]. Among these complications, cardiac autonomic neuropathy (CAN) is particularly concerning. CAN involves dysfunction within the autonomic nervous system, primarily affecting the heart and vascular system, and is frequently linked to diabetes mellitus [2]. Effective management of T2DM and its complications, particularly CAN, is crucial for improving patient outcomes and quality of life (QoL). 

Exercise training has been established as a cornerstone of diabetes management due to its beneficial effects on glycemic control, cardiovascular health, and overall fitness [3]. Both aerobic and resistance training are recognized as crucial interventions for addressing T2DM and its related complications [3,4]. Aerobic exercise has been well-documented to enhance insulin sensitivity and improve glucose utilization in individuals with T2DM, thereby improving glycemic control [5,6]. These benefits are underpinned by mechanisms such as enhanced GLUT4 expression and improved mitochondrial function, which aid in the uptake and utilization of glucose by skeletal muscles [7,8]. 

Resistance training, aimed at muscle strengthening and hypertrophy, has been demonstrated to improve insulin action and metabolic regulation through muscle growth and increased muscle capacity for glucose uptake [8,9]. This form of exercise significantly alters body composition, promoting an increase in lean mass while decreasing fat mass [10]. Despite the well-known benefits of aerobic and resistance training, the optimal integration of these modalities for individuals with T2DM, particularly those with CAN, remains unclear.

Concurrent exercise training (CET), which incorporates both endurance and strength training in the same workout routine, provides the combined advantages of each type of exercise [11]. This approach has shown promise in significantly enhancing glycemic control, body composition, cardiorespiratory fitness (CRF), lipid profile, inflammatory markers, and insulin sensitivity in this population [12]. The combined effects of concurrent training might also enhance cardiac autonomic function and reduce the risk of CAN, thereby addressing major concerns related to T2DM and its complications.

Cardiac autonomic modulation, which reflects the balance between sympathetic and parasympathetic nervous activity, is a critical indicator of cardiovascular health [13]. Improved autonomic function is associated with better cardiovascular outcomes and reduced mortality in individuals with T2DM [14]. Recent studies have demonstrated that low levels of cardiac autonomic function as indicated by heart rate variability (HRV), are correlated with low CRF, measured by maximum oxygen consumption (VO_2max_), in individuals with T2DM [15].

Body composition and CRF are key determinants of health in individuals with T2DM [16]. CET has been shown to improve these parameters by reducing percentage body fat (PBF), increasing lean muscle mass, and enhancing aerobic capacity [12,17]. Additionally, these improvements contribute to a better metabolic profile, as reflected by improved glycemic control and lipid profiles [18]. Moreover, these improvements can lead to better QoL by reducing diabetes-related symptoms and enhancing physical and mental well-being [19].

Despite the potential benefits, the impact of CET on cardiac autonomic modulation in patients with T2DM and CAN has not been thoroughly investigated. Previous studies have predominantly focused on either aerobic or resistance training alone, with limited data on the synergistic effects of combined modalities [20,21]. Moreover, the comprehensive evaluation of exercise interventions on a spectrum of outcomes, including HRV, heart rate recovery (HRR), metabolic profile, body composition, CRF, and QoL, remains scarce.

This randomized controlled trial aims to fill this gap by examining the effects of a structured CET program on various health parameters in individuals with T2DM and CAN. The primary objective is to assess changes in cardiac autonomic modulation as measured by HRV indices and HRR measures. Secondary objectives include evaluating the impact on metabolic profile, body composition, CRF, and QoL.

By addressing these multifaceted outcomes, this study seeks to provide a robust evidence base for the incorporation of CET in the management of T2DM patients with CAN. The findings are expected to enhance clinical guidelines and offer practical insights for healthcare providers aiming to optimize the therapeutic strategies for this high-risk population.

## 2. Materials and Methods

### 2.1. Subjects and Settings

Participants for this study were recruited from 1 October 2021 to 28 December 2023, with the study conducted collaboratively at the Dr. M.A. Ansari Health Centre and the Centre for Physiotherapy and Rehabilitation Sciences (CPRS) at Jamia Millia Islamia. Recruitment was facilitated through referrals from the Dr. M.A. Ansari Health Centre, where initial assessments by medical professionals determined participant eligibility. The trial was registered prospectively in the Clinical Trials Registry—India (CTRI/2021/09/036711) and received ethical approval from the Institutional Ethics Committee at Jamia Millia Islamia (24/5/323/JMI/IEC/2021). Each participant was provided with an information sheet explaining the study’s purpose, methodology, and rights, and they provided written informed consent. All procedures adhered to the Declaration of Helsinki and its subsequent amendments [22].

Eligibility criteria included adults aged between 35 and 70 years with a diagnosis of T2DM for more than one year, confirmed based on the American Diabetes Association (ADA) criteria by qualified medical professionals [23]. The duration of diabetes was evaluated through a thorough screening and detailed history taking. Participants had a sedentary lifestyle, defined as less than one hour of exercise per week in the previous six months, and the presence of CAN, either early or definite, as per the Ewing criteria [24]. For the diagnosis of CAN cardiac autonomic reflex testing (CARTs) was employed using the Ewing battery method during the screening process. This includes tests for parasympathetic and sympathetic functions. For parasympathetic function, the deep breathing test measures the expiration-to-inspiration ratio (E/I), the head-up tilt (HUT) test determines the 30:15 ratio, and the Valsalva maneuver calculates the Valsalva ratio (VR). For sympathetic function, the blood pressure response to standing and the sustained handgrip test were conducted. Participants were categorized as CAN-positive or CAN-negative based on the number of abnormalities in parasympathetic and sympathetic tests. CAN-positive individuals were further classified into early CAN (one abnormal parasympathetic result), definite CAN (two or more parasympathetic abnormalities), and severe CAN (abnormalities in both parasympathetic and sympathetic tests) according to Ewing’s criteria [24]. 

Participants were excluded if they had any major co-existing cardiac disease, were on exogenous insulin, engaged in moderate to vigorous physical activity for more than 60 min per week, or were on β-blocker therapy. Additional exclusion criteria included retinopathy or nephropathy, musculoskeletal abnormalities that could preclude the intervention, and contraindications to exercise stress testing as outlined by the American College of Sports Medicine (ACSM) guidelines. These ACSM contraindications included significant changes in resting ECG suggesting ischemia, recent myocardial infarction or other acute cardiac events, unstable angina, uncontrolled cardiac arrhythmias, severe symptomatic aortic stenosis, uncontrolled symptomatic heart failure, acute pulmonary embolus, acute myocarditis or pericarditis, and acute infections [25].

### 2.2. Experiment and Procedure

The sample size for this randomized controlled trial (RCT) was determined using G*Power (version 3.1.9) based on significant changes in HbA1C levels post-exercise as reported by a previous study [26]. To achieve an effect size of 0.95 with an alpha level of 0.05 and a power of 0.95, a sample of 60 participants (30 per group) was required. Considering a 37% dropout rate, the total sample size was increased to 96 participants, resulting in 48 participants per group.

A thorough screening process using a structured form was conducted for each subject, followed by baseline assessments to establish starting points for all participants. Participants were then randomly assigned to either the Interventional Group, which received CET three times per week, or the Control Group, which received usual care without additional interventions. All criterion measures were reassessed after 13 weeks to evaluate changes and outcomes.

On the first day of assessment, a trained professional collected 2 mL of blood from each participant’s antecubital vein to analyze key health markers such as glycemic and lipid profiles. Participants also completed questionnaires assessing their QoL. On a subsequent day, comprehensive examinations were conducted to assess cardiac autonomic function, cardiovascular health, VO_2max_, and body composition.

To maintain confidentiality, all identifying information on consent forms and demographic/injury history questionnaires was securely stored, and each participant was assigned a unique identifier number to ensure anonymity throughout the study. Participants were advised to refrain from engaging in any structured exercise regimes other than their routine physical activities during the study period to ensure that the effects of the CET program could be isolated and accurately measured.

The randomization process employed block randomization to allocate eligible participants into either the interventional or control groups. This involved creating a computer-generated randomized number sequence using Microsoft Excel 2013 with the syntax fx = RAND(). Blocks were constructed to contain an equal number of participants in each group, maintaining a 1:1 ratio. The allocation sequence was concealed in serially numbered, opaque envelopes. When a participant was eligible and consented to join the study, the next sequential envelope was opened to assign the participant to the indicated group.

The study was initially designed as a single-blind trial where participants were unaware of their group allocation. However, due to the nature of the exercise intervention, participants were inherently aware of their group assignment. Assessors and intervention providers were not blinded to the group assignments. To mitigate assessment bias, performance bias, and detection bias, the study incorporated several strategies. These included the standardization of procedures, where all assessments and interventions were conducted following strict, standardized protocols, and comprehensive training for assessors and intervention providers to ensure adherence to protocols and maintain objectivity.

### 2.3. Evaluation

#### 2.3.1. Assessment of Cardiac Autonomic Function

Evaluations of cardiac autonomic function were scheduled during the morning hours, from 9:00 to 12:00 AM, to control diurnal variations in autonomic activity. Participants were also required to fast for at least 2 h before testing to avoid the influence of digestion on the results [27,28]. To minimize the impact of medications on autonomic nervous system measures, participants were instructed to refrain from taking any drugs that could affect the system, such as beta-blockers, for 24 h before the tests [29,30]. Additionally, participants were asked to avoid consuming substances like caffeine and nicotine for 12 h and to refrain from physical activity for 24 h before the tests. These precautions help to eliminate any temporary effects these factors might have on the autonomic nervous system, ensuring more reliable and consistent results [27,28,31].

- Resting Heart Rate and Heart Rate Variability Assessment

Participants were assessed for resting heart rate (HR_rest_) and HRV after resting for at least 15 min in a supine position under controlled environmental conditions set at 24 °C. Electrocardiograph (ECG) recordings were taken for 10 min using the standard lead II configuration, with the last 5 min of this recording used for detailed analysis of HRV’s time and frequency domain variables. The data were recorded and processed using Lab Chart software version 7.3.7 (Power Lab 8 SP, AD Instruments, Bella Vista NSW, Australia).

ECG data were carefully examined to identify any ectopic beats. If ectopic beats were found, they were interpolated to maintain a continuous data stream, provided they accounted for less than 10% of the total beats. Power spectral analysis was conducted using the Fast Fourier Transform (FFT), which separated the signal power into various frequency components. Data filtering was performed with a lowpass filter set at a cut-off frequency of 40 Hz.

The analysis evaluated several metrics, including Total Power (TP), low-frequency (LF) power, high-frequency (HF) power in milliseconds squared (ms^2^), and the LF/HF ratio. Time domain variables such as the standard deviation of N-N intervals (SDNN), the root mean square of successive differences between adjacent R-R intervals (RMSSD), and the percentage of consecutive R-R intervals differing by more than 50 milliseconds (pNN50) were also analyzed. Both data acquisition and analysis adhered to the standards established by the Task Force of the European Society of Cardiology and the North American Society of Pacing and Electrophysiology (1996), ensuring the accuracy and reliability of the HRV measurements [29].

- Heart Rate Recovery Assessment

In this study, heart rate recovery (HRR) was assessed to evaluate cardiac autonomic function. HRR reflects the rate at which the heart rate declines after peak exercise and is an important indicator of parasympathetic reactivation and sympathetic withdrawal [32]. The assessment was conducted following a standardized exercise test using the Modified Bruce Protocol, a well-established protocol designed to incrementally increase exercise intensity and safely determine the maximal exercise capacity of participants [33].

Participants began the exercise test with a warm-up phase at a low intensity, gradually increasing the intensity every three minutes until they reached their peak heart rate (HRpeak). This incremental approach helped ensure participant safety and allowed for accurate determination of maximal cardiovascular effort. Upon reaching HRpeak, participants immediately ceased exercise and transitioned to a recovery phase in a supine position.

During the recovery phase, heart rate measurements were recorded at multiple intervals: 30 s (HR30sec), 1 min (HR1min), 2 min (HR2min), and 3 min (HR3min) post-exercise. These time intervals were selected based on established protocols for evaluating HRR, which capture the progressive decline in heart rate due to autonomic modulation [24,34].

The HRR at each time interval was calculated by subtracting the heart rate at each respective time point from the HR_peak_. Specifically, HRR30sec was calculated as HR_peak_ minus HR30sec, HRR1min as HR_peak_ minus HR1min, HRR2min as HR_peak_ minus HR2min, and HRR3min as HR_peak_ minus HR3min. These calculations provided a quantitative measure of the rate at which heart rate returned to baseline levels post-exercise, thus reflecting the efficiency of cardiac autonomic recovery.

#### 2.3.2. Blood Pressure 

Blood pressure measurements were taken using a standard sphygmomanometer. To ensure accuracy, participants were instructed to avoid caffeine, smoking, and physical exercise for at least 30 min before the assessment. They sat calmly for 5 min and then rested in a seated position for an additional 10 min. Blood pressure was measured on either the right or left arm, and the initial reading was recorded. After a 2 min interval, a second measurement was taken on the same arm, following the protocol recommended by the American Heart Association [35]. Subsequently, a second set of measurements was taken from the opposite arm after a brief 1 to 2 min rest. If there was a variation between the two arms, the higher reading was used for analysis [36].

#### 2.3.3. Body Composition Assessment

Anthropometric measurements included height and weight to calculate the Body Mass Index (BMI) [37]. Waist circumference was measured at the midpoint between the iliac crest and the lower margin of the floating ribs during normal expiration [38], while hip circumference was measured at the level of the maximal gluteal protuberance [39]. These measurements were used to determine the Waist-to-Hip Ratio (WHR). Skinfold thickness was measured at specific sites using a Harpenden Skinfold Caliper (Galaxy Informatics, India). For men, measurements were taken at the chest, abdominals, and thigh; for women, at the triceps, suprailiac, and thigh. These skinfold measurements were used to calculate body fat percentage (PBF) using established equations from previous research [40,41].

#### 2.3.4. Biochemical Assessments

Blood samples were collected from participants following an overnight fast. A trained medical technician used an indwelling cannula to draw 2–5 mL of blood from the antecubital vein while participants were seated. These samples were analyzed to assess various biochemical parameters.

The lipid profile, including total cholesterol, triglycerides, high-density lipoprotein cholesterol (HDL-C), and low-density lipoprotein cholesterol (LDL-C), was measured using an enzymatic colorimetric technique with Span Cogent Diagnostic kits. Fasting plasma glucose and postprandial blood glucose levels were determined using the glucose oxidase (GOD) method (Span Cogent Diagnostics, GOD–POD). In glycemic control, levels of fasting blood glucose (FBG), postprandial blood glucose (PPG), and HbA1c were measured from whole blood samples using a commercial kit (Crystal Chem., 80099), where samples were lysed in the presence of proteases to facilitate analysis. 

#### 2.3.5. Quality of Life

The assessment of QoL was conducted using the Audit of Diabetes-Dependent QoL (ADDQoL-19) questionnaire, an updated form of the original tool created by Bradley et al. in 1999 [42,43]. The license for the ADDQoL-19 (Hindi version) was granted by Health Psychology Research Ltd., High St. Egham, UK (https://healthpsychologyresearch.com), with the license number CB1325. Participants were asked to complete the ADDQoL-19 questionnaire themselves. The ADDQoL-19 begins with two initial items that evaluate general QoL (G-QoL) and diabetes-dependent QoL (DD-QoL). The first item, assessing G-QoL, is rated on a seven-point Likert scale ranging from “excellent” to “extremely bad”. The second item, assessing DD-QoL, uses a five-point Likert scale ranging from “very much better” to “worse”.

Following these initial items, the questionnaire consists of 19 domains specifically related to diabetes-dependent QoL. Each domain includes a sub-domain that assesses the importance of that aspect of QoL. The main domain scale is graded on a 5-point scale from -3 (maximum negative impact of diabetes) to 1 (maximum positive impact), while the sub-domain importance scale ranges from 3 (very important) to 0 (not at all important). The Weighted Impact Score (WIS) for each domain is calculated by multiplying the importance and impact scores, resulting in values ranging from −9 to +3. A lower WIS indicates a poorer QoL in that domain. To obtain an overall measure, the Average Weighted Impact Score (AWIS) is derived by averaging all the domain WIS values.

Participants were provided with the ADDQoL-19 questionnaire and instructed to complete it independently. The questionnaire was distributed to participants during their visit to the clinic both at baseline and after the intervention. Clear instructions were given to ensure participants understood how to complete the questionnaire, including information on the scales and how to rate each item. Participants filled out the questionnaire in a quiet and comfortable environment, ensuring privacy and minimizing distractions. After completion, the questionnaires were collected and checked for completeness. This self-administered approach ensures that participants provide their own perspectives on their QoL without external influence, leading to more accurate and personal reflections on how diabetes impacts their daily lives.

#### 2.3.6. Cardiorespiratory Fitness

The CRF in this study was evaluated by measuring VO_2max_ using a direct breath-by-breath analysis method. Participants breathed through a mouthpiece connected to a within an open circuit spirometry system. This setup facilitated the accurate collection of respiratory data, which was analyzed using the Metabolic Module in LabChart Software (version 8; AD Instruments). This method ensures precision and reliability in VO_2max_ measurements, adhering to established protocols for assessing aerobic fitness [44].

The modified Bruce protocol was employed for the graded exercise test on a treadmill, beginning at a lower intensity and progressing through stages of increased speed and incline every 3 min. This approach is suitable for a wide range of fitness levels, ensuring participants can safely reach their maximum exertion [45]. Continuous monitoring of heart rate, oxygen uptake, and expired gases throughout the test provided real-time feedback, essential for the accurate calculation of VO_2max_ and a comprehensive assessment of CRF.

### 2.4. Training Program

#### 2.4.1. Concurrent Exercise Training

Before initiating each exercise session, a comprehensive pre-training assessment was conducted to ensure the safety and efficacy of the training for each participant. This evaluation included three critical measurements: blood glucose monitoring, resting blood pressure and heart rate assessment, and hypoglycemia management [46,47]. Participants’ blood glucose levels were meticulously checked upon arrival. If a participant’s blood glucose was below 100 mg/dL, a snack containing 15 g of carbohydrates was provided to mitigate the risk of hypoglycemia during exercise. Conversely, if a participant’s pre-exercise blood glucose exceeded 300 mg/dL, they were still permitted to commence their exercise regimen, but a reevaluation was conducted after 20 to 30 min to monitor any further elevation in blood glucose levels [48]. Should there be an increase, the exercise session immediately ceased to prevent adverse health effects. Alongside blood glucose monitoring, resting blood pressure and heart rate were recorded to understand the subject’s baseline cardiovascular status, which could influence the safety and intensity of the prescribed exercise. The protocol was particularly vigilant about the possibility of hypoglycemia. 

The concurrent training program implemented in the study consisted of aerobic and resistance exercises carefully designed to adhere to the joint guidelines set forth by the ACSM and the ADA. This integration of guidelines also aligns with the practical recommendations delineated in the ACSM’s guidance documents [49].

- Aerobic Training

The aerobic component of the program was executed through treadmill walking, with a deliberate and systematic escalation in both intensity and duration. Participants began their training with an intensity range from 40% to 65% of their heart rate reserve, calculated as the difference between maximum heart rate and resting heart rate [50], and continued for 30 to 60 min. During the initial two weeks, the emphasis was on extending the duration of the aerobic activity by approximately 5 to 10 min, contingent upon each participant’s response. Subsequently, for the remaining 13 weeks, the focus alternated between increasing the duration and intensity, with the latter increasing by about 10% of the heart rate reserve every 4 weeks [51].

- Resistance Training

The resistance training was designed around eight core exercises targeting both upper and lower body muscles, including the leg press, knee flexion, knee extension, chest press, lat pull-down, shoulder press, abdominal crunches, biceps curls, and triceps press down. The resistance training protocol spanned a 13-week period divided into five phases [52]. During weeks 1–2, participants performed 12–15 repetitions at 60–65% of one-repetition maximum (1-RM) with sessions occurring thrice weekly. Weeks 2–4 continued with the same repetition range and intensity as the first phase, maintaining the thrice-weekly session frequency. In weeks 4–6, the intensity increased, with participants performing 8–10 repetitions at 70% of 1-RM, also three times per week. This intensity and repetition range were maintained through weeks 6–8 and weeks 8–13, with thrice-weekly sessions sustained. Muscle strength was assessed using the 1-RM for both upper and lower body exercises, estimated using the Brzycki equation based on the weight lifted and the number of repetitions completed [53]. 

#### 2.4.2. Control Group

The control group received standard care, which included conventional medical treatment and the continuation of their daily routine activities. No supervised exercise training was offered to patients in the control group. Participants in both the exercise and control groups were given general dietary instructions suitable for individuals with T2DM, based on the ADA guidelines [54]. The dietary instructions were similar in both groups, and no additional diet recommendation was provided during the intervention period. Participants were also instructed to maintain their usual dietary habits and were not encouraged to follow any specific diet. Compliance was monitored by having participants maintain a diary of their daily meals, which was checked during patient visits.

### 2.5. Statistical Analyses

Statistical analysis was performed using IBM SPSS version 27 (IBM SPSS, Statistics, Chicago, IL, USA), with significance set at *p* < 0.05. The normality of the dataset was assessed using the Shapiro–Wilk test. Baseline differences between the CET group and the control group were analyzed using independent *t*-tests for continuous variables and chi-square tests for categorical variables. We also employed a 2 × 2 mixed ANOVA to examine the main effects and interactions of group (CET vs. control) and time (baseline vs. post-test).

## 3. Results 

### 3.1. Participant Flow

The final analysis of our randomized controlled trial used an intention-to-treat (ITT) approach, with the last observation carried forward method to include participants who dropped out or deviated from the protocol. This ensured all randomized participants were analyzed in their originally assigned groups [55]. 

**Figure 1:**
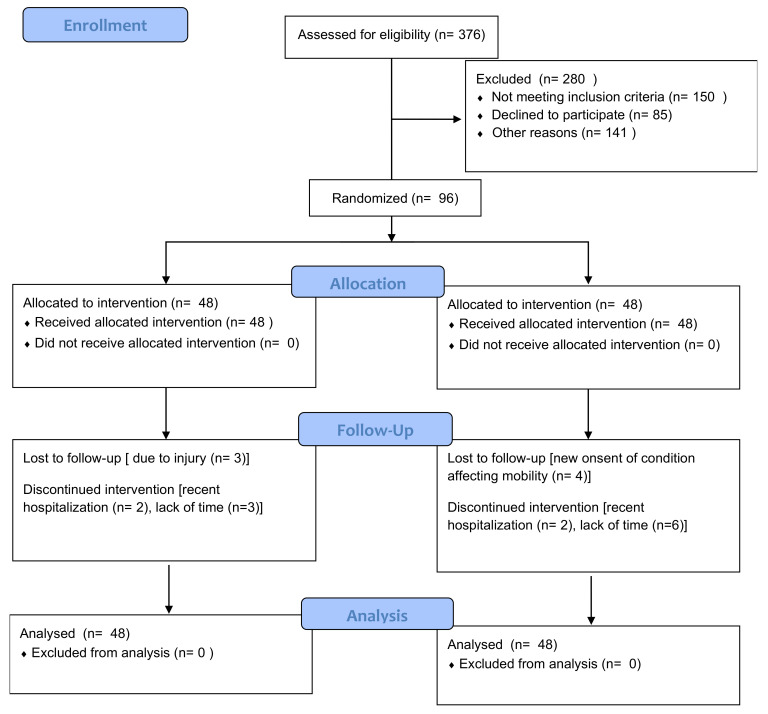
Consolidated Standards of Reporting Trials (CONSORT) flow diagram.

A total of 376 participants were assessed for eligibility. Of these, 280 were excluded for various reasons: 150 did not meet the inclusion criteria, 85 declined to participate, and 45 were excluded for other reasons. Consequently, 96 participants were randomized into two groups, with 48 participants allocated to the CET group and 48 to the control group. All participants in each group received the allocated intervention, with no participants missing the intervention in either group. In the CET group, three participants were lost to follow-up due to injury, and five participants discontinued the intervention due to recent hospitalization (two participants) and lack of time (three participants). In the control group, four participants were lost to follow-up due to the new onset of a condition affecting mobility, and eight participants discontinued the intervention due to recent hospitalization (two participants) and lack of time (six participants). All 48 participants in each group were included in the final analysis, with no participants excluded from the analysis. Despite the dropouts, the ITT approach ensured their data were included in the final analysis, maintaining the study’s randomization integrity (Figure 1).

### 3.2. Baseline Characteristics

The baseline characteristics of the participants are summarized in Table 1. No significant differences were found between the CET and control groups regarding age, sex, marital status, professional activity, education level, place of residence, smoking and alcohol habits, duration of diabetes, type of antidiabetic treatment, and CARTs parameters. This indicates that the two groups were comparable at the start of the intervention (Table 1).

Table 2 presents the comparison of primary and secondary outcome measures between the groups at baseline. There were no significant differences in resting HRV parameters heart rate recovery cardiovascular function, glycemic control, body composition, lipid profile, cardiorespiratory fitness, and QoL measures between the CET and control groups at baseline. This confirms that the initial status of participants in both groups was similar (Table 2).

### 3.3. ANOVA Findings

The 2 × 2 mixed ANOVA revealed significant main effects of time, group, and group × time interactions for most measured outcomes, underscoring the efficacy of the CET program (Table 3).

**Figure 2:**
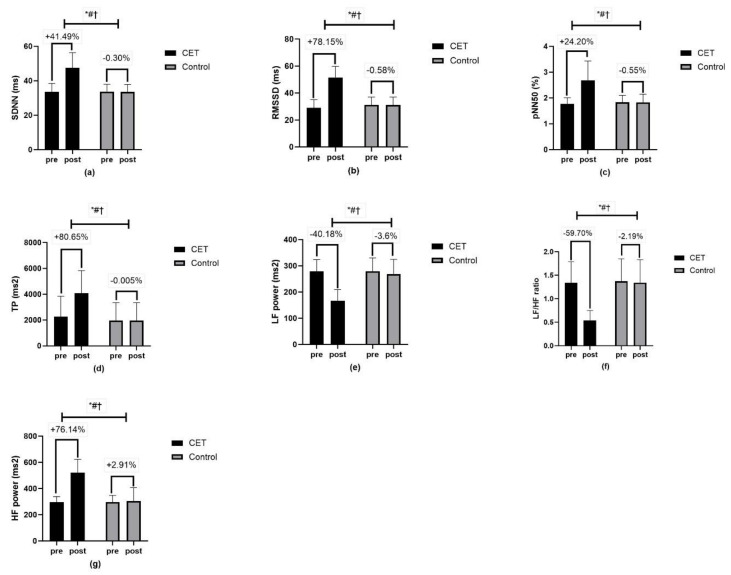
Resting heart rate variability parameters (**a**–**e**): (**a**) SDNN-standard deviation of N-N intervals, (**b**) RMSSD-root mean square of successive differences between adjacent R-R intervals, (**c**) pNN50-Proportion of differences in consecutive N-N intervals that are longer than 50 ms, (**d**) TP—total power (ms^2^), (**e**) LF-low frequency, (**f**) HF-high frequency, (**g**) LF/HF ratio-ratio of low- and high-frequency power; ms-milliseconds. Data are presented as mean ± standard deviation; * significant group effect; # significant interaction effect; † significant time effect.

**Figure 3:**
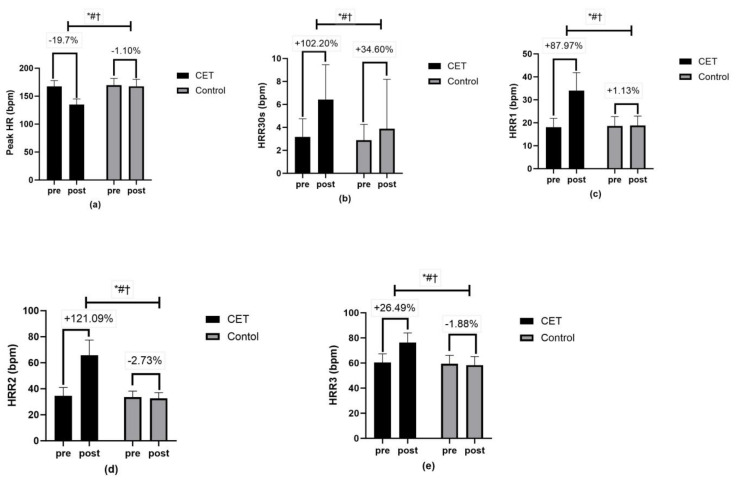
Heart rate recovery (HRR) (**a**–**e**): (**a**) Peak heart rate, (**b**) HRR in first 30 s, (**c**) HRR in first minute, (**d**) HRR in second minute, (**e**) HRR in first minute; * significant group effect; # significant interaction effect; † significant time effect.

HRV metrics such as SDNN, RMSSD, pNN50, TP, low-frequency LF power, HF power, and the LF/HF ratio all showed significant effects (*p* < 0.01) with high effect sizes (partial eta squared, pη^2^ ranging from 0.16 to 0.68). Further, HRR metrics such as peak HR, HRR30s, HRR1, HRR2, and HRR3 also showed significant effects (*p* < 0.01) for time, group, and group × time interactions, indicating improvements in cardiac autonomic function (Figure 2 and Figure 3).

**Figure 4:**
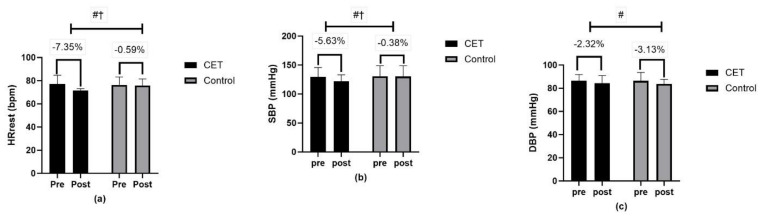
Cardiovascular risk factors (**a**–**c**): (**a**) HRrest-Resting heart rate, (**b**) SBP-Systolic blood pressure, (**c**) DBP-Diastolic blood pressure; # significant interaction effect; † significant time effect.

**Figure 5:**
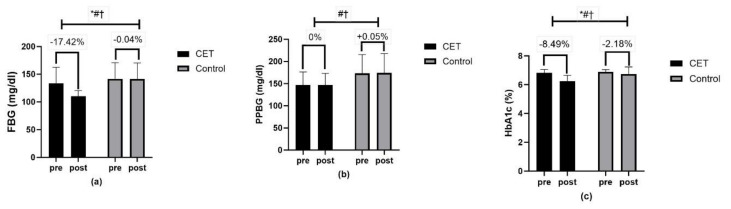
Glycemic control (**a**–**c**): (**a**) FBG-fasting blood glucose; (**b**) PPBG-post-prandial blood glucose; (**c**) HbA1c-glycosylated hemoglobin; * significant group effect; # significant interaction effect; † significant time effect.

Cardiovascular function measures, including HR_rest_ and systolic blood pressure (SBP), displayed significant time effects, and SBP showed a significant group x time interaction (Figure 4). Moreover, in the glycemic profile, a significant effect was demonstrated of time, group, and group x time interaction for FBG and HbA1c (Figure 5).

**Figure 6:**
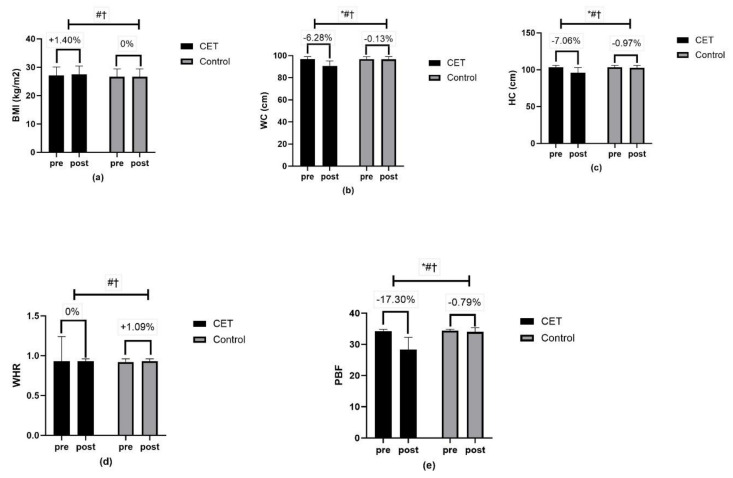
Body composition marker; (**a**) BMI-body mass index; (**b**) WC-Waist circumference; (**c**) HC-Hips circumference; (**d**) WHR-Waist–hip ratio; (**e**) PBF-percentage body fat; * significant group effect; # significant interaction effect; † significant time effect.

**Figure 7:**
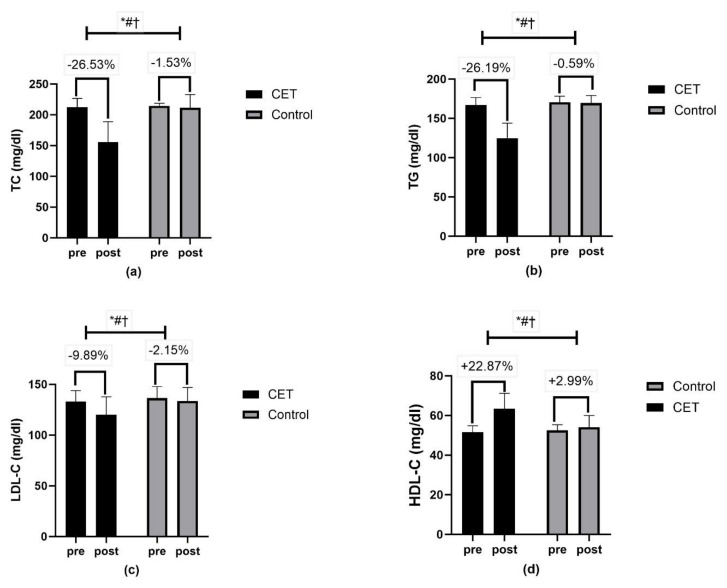
Lipid profile (**a**–**d**): (**a**) TC- total cholesterol; (**b**) TG-triglycerides; (**c**) LDL-C-low-density lipoprotein cholesterol; (**d**) HDL-C-high-density lipoprotein cholesterol; * significant group effect; # significant interaction effect; † significant time effect.

**Figure 8:**
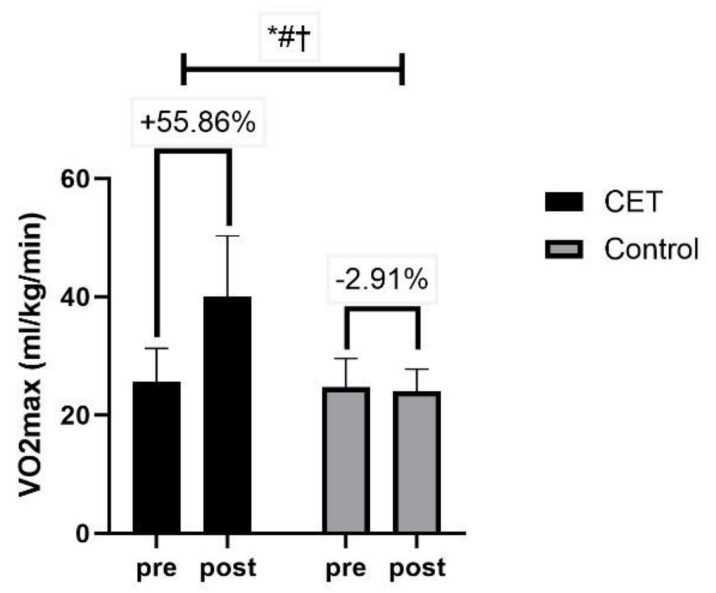
Cardiorespiratory fitness using VO_2max_ (volume of maximum oxygen consumption); * significant group effect; # significant interaction effect; † significant time effect.

**Figure 9:**
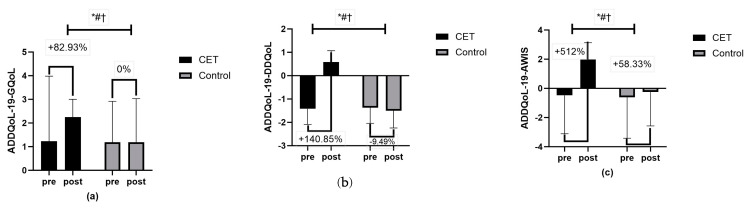
ADDQoL-19: 19-item Audit of Diabetes Dependent Quality of Life; (**a**) ADDQ0L-19 GQoL- general quality of life, (**b**) ADDQoL-19 DDQoL-diabetes-dependent quality of life, (**c**) ADDQoL-19 AWIS-average weighted impact score; * significant group effect; # significant interaction effect; † significant time effect.

Body composition measures, including waist circumference, hip circumference, and PBF, revealed significant time, group, and group x time interactions (*p* < 0.01) (Figure 6). Lipid profile measures, such as total cholesterol, triglycerides, LDL-C, and HDL-C, showed significant effects of time, group, and group x time interactions (*p* < 0.01) (Figure 7). The CRF (Figure 8) and QoL measures (Figure 9) also showed significant improvements with substantial interaction effects (*p* < 0.01), highlighting the broad benefits of the CET program for individuals with T2DM and CAN.

## 4. Discussion

The findings of this RCT demonstrate that CET significantly enhances multiple health parameters in individuals with T2DM and CAN. These parameters include cardiac autonomic modulation, metabolic profile, body composition, CRF, and QoL. The comprehensive assessment provided in this study underscores the multifaceted benefits of CET, offering valuable insights for clinical practice and patient management.

### 4.1. Cardiac Autonomic Modulation

The primary outcome of this study was to assess the changes in cardiac autonomic modulation as measured by HRV indices. The CET group exhibited significant improvements in HRV parameters, including SDNN, RMSSD, pNN50, TP, LF power, HF power, and LF/HF ratio. These changes indicate enhanced parasympathetic activity and a more balanced autonomic function. These findings align with a previous study suggesting that regular exercise, particularly when combining aerobic and resistance training, can improve autonomic function in individuals with T2DM [56]. These enhancements in HRV can be linked to several mechanisms, including increased parasympathetic activity due to aerobic exercise, decreased sympathetic dominance resulting from resistance training, enhanced baroreflex sensitivity, and the modulation of neuroendocrine factors. Collectively, these changes contribute to improved autonomic balance [57,58]. Moreover, the improved HRV is associated with better cardiovascular health and reduced mortality risk in diabetic patients [59]

The results of our study demonstrated significant improvements in HRR among participants in the CET group compared to the control group. Specifically, measures such as HRR30s, HRR1, HRR2, and HRR3 all showed substantial enhancement, indicating a positive shift in cardiac autonomic modulation [60]. These findings align with existing literature, underscoring HRR as a robust marker of autonomic function and a predictor of cardiovascular health [61]. Improvements in HRR are largely attributable to increased vagal tone and reduced sympathetic activity post-exercise, which together enhance parasympathetic reactivation and accelerate heart rate deceleration [62].

The physiological mechanisms underlying these improvements include enhanced vagal activity, reduced sympathetic dominance, improved CRF, and beneficial neural remodeling [15,60]. Regular exercise training promotes these changes by fostering a more efficient autonomic response and improving overall cardiovascular health [21,63]. The importance of these results is evident in their clinical implications, suggesting that customized exercise programs that combine aerobic and resistance activities can effectively counteract autonomic dysfunction in T2DM patients with CAN, potentially lowering their risk of cardiovascular disease.

### 4.2. Metabolic Profile

The findings from our study indicate that CET significantly enhances the metabolic profile of individuals with T2DM and CAN. Specifically, participants in the CET group exhibited substantial reductions in fasting blood glucose, postprandial blood glucose, and HbA1c levels. These results align with an earlier systematic review with a meta-analysis that revealed the advantages of CET in improving insulin sensitivity and glycemic control [12]. The enhancement in glycemic control can primarily be credited to the synergistic impact of aerobic and resistance exercises on insulin sensitivity and glucose metabolism. Aerobic exercise enhances GLUT4 expression and mitochondrial functionality, leading to improved glucose uptake by muscle cells [7]. Concurrently, resistance training increases muscle mass, which boosts the muscles’ capacity for glucose storage and usage [9]. These physiological changes not only enhance short-term glucose regulation efficiency but also may help stabilize blood sugar levels over the long term [64].

In addition to glycemic control, the CET group showed significant improvements in lipid profile markers, including reductions in total cholesterol, triglycerides, LDL-C, and an increase in HDL-C. These changes are indicative of a lower cardiovascular risk and better overall metabolic health. Aerobic exercise has been well-documented to improve lipid metabolism, while resistance training enhances muscle mass and insulin action, collectively contributing to these beneficial outcomes [65]. By managing hyperglycemia with non-pharmacological methods, concurrent training provides an effective strategy for controlling T2DM, potentially decreasing reliance on medication and lessening the risk of complications linked to inadequate glycemic control. Overall, the results of this study highlight the comprehensive benefits of CET in improving the metabolic profile of individuals with T2DM and CAN, supporting the integration of structured exercise programs in routine management strategies for T2DM. Future research should explore the long-term sustainability of these metabolic improvements and the potential for CET to further reduce cardiovascular events in this high-risk population.

### 4.3. Body Composition

The results of our study demonstrated significant improvements in body composition among participants in the CET group compared to the control group. Specifically, the CET group showed notable reductions in waist circumference, hip circumference, and PBF. These findings align with previous studies indicating that concurrent aerobic and resistance training is effective in reducing adiposity and improving overall body composition in individuals with T2DM [26,52]. The reduction in waist circumference and hip circumference is particularly important as these metrics are strongly associated with cardiovascular risk and metabolic health [51,66].

These results are consistent with available literature that highlights the benefits of both aerobic and resistance training in altering body composition [12,67]. Aerobic exercise promotes fat oxidation and energy expenditure, leading to reductions in body fat, while resistance training increases muscle mass, which in turn enhances basal metabolic rate and improves glucose utilization [48]. The synergistic effects of CET on body composition can be attributed to several physiological mechanisms. Aerobic exercise enhances mitochondrial density and function, promoting fat oxidation and reducing adiposity [68]. Concurrently, resistance training induces muscle hypertrophy and increases the capacity for glucose storage and utilization, thereby improving insulin sensitivity [69]. These changes not only improve physical appearance and fitness but also contribute to better metabolic health and reduced risk of diabetes-related complications. The observed improvements in body composition metrics in our study underscore the effectiveness of CET as a comprehensive strategy for managing T2DM and enhancing overall health outcomes.

### 4.4. Cardiorespiratory Fitness

This study demonstrated that CET significantly enhances cardiorespiratory fitness CRF in individuals with T2DM and CAN. The metric for assessing CRF in this study was VO_2max_, which showed a substantial increase in the CET group compared to the control group. This improvement in VO_2max_ is statistically significant, underscoring the efficacy of CET in enhancing aerobic capacity.

These findings align with previous research demonstrating the positive impact of combined aerobic and resistance training on VO_2max_ in diabetic populations [26,52]. Further, a recent study by Zaki et al. (2024) found strong positive correlations between VO_2max_ and HRV metrics, indicating that higher CRF levels are associated with enhanced parasympathetic modulation and reduced sympathetic dominance in T2DM patients [15]. This relationship underscores the intricate link between autonomic function and aerobic capacity, suggesting that improvements in CRF through CET can lead to better overall cardiovascular health.

The physiological mechanisms underlying these improvements include enhanced cardiovascular efficiency, primarily through increased cardiac output and capillary density in muscle tissues, which facilitate greater oxygen delivery and extraction during aerobic exercise [70,71]. Additionally, resistance training contributes to improving muscular strength and endurance, which can lead to more efficient muscle oxygen utilization and increased mitochondrial density [71]. These adaptations not only enhance VO_2max_ but also improve the metabolic control of glucose, which is critically important for managing diabetes [12].

Moreover, the improvements in CRF observed in this study also align with enhanced autonomic function as indicated by HRV metrics. Improved autonomic function is associated with better cardiovascular health and reduced mortality risk in diabetic patients [59]. The enhanced parasympathetic activity and reduced sympathetic dominance resulting from CET contribute to a more balanced autonomic function, which supports overall cardiovascular health [58].

### 4.5. Quality of Life

The study’s findings demonstrate that CET significantly improves various aspects of QoL in individuals with T2DM and CAN. The impact on QoL was assessed using the ADDQoL-19 questionnaire, which measures G-QoL, DD-QoL, and AWIS.

The results showed a notable improvement in the G-QoL for the CET group compared to the control group. After the intervention, the CET group reported a significantly higher G-QoL score. This suggests that the CET program contributed substantially to the enhancement of the general QoL among the participants. This improvement can be attributed to the comprehensive nature of CET, which includes both aerobic and resistance training, leading to better physical and psychological well-being. Further, the DD-QoL score also exhibited significant improvements. The CET group showed a positive score post-intervention, in contrast to a negative score in the control group. This underscores the effectiveness of CET in enhancing diabetes-specific aspects of QoL. This improvement in DD-QoL is critical as it directly relates to how diabetes impacts daily living and emotional well-being, reflecting a holistic enhancement in the patient’s perception of their health and life satisfaction. The AWIS also demonstrated significant improvements in the CET group compared to the control group. The AWIS measures the overall impact of diabetes on various life domains, and the positive change indicates that the structured CET program effectively mitigates the negative impact of diabetes on patients’ lives. This score integrates various dimensions of QoL, providing a comprehensive assessment of the intervention’s benefits.

These improvements can be attributed to the multifaceted benefits of CET, which include better glycemic control, enhanced cardiovascular fitness, and improved mental health. Enhanced insulin sensitivity and reduced inflammation from regular exercise contribute to better physical health and reduced diabetes-related complications [12,51]. Mechanistically, aerobic exercise increases endorphin levels and reduces stress hormones, thereby improving mood and mental well-being [72]. Resistance training, on the other hand, enhances muscle strength and endurance, leading to better physical functioning and reduced fatigue [72,73]. These physiological changes collectively contribute to improved QoL as patients experience fewer diabetes-related symptoms and greater overall well-being.

### 4.6. Clinical Utility in Daily Practice

In addition to demonstrating the significant benefits of CET on various health metrics in individuals with T2DM and CAN, our findings hold considerable clinical utility for daily practice. Implementing structured CET programs in clinical settings can provide a non-pharmacological intervention that effectively improves cardiac autonomic modulation, metabolic profile, body composition, CRF, and QoL. Healthcare providers can incorporate CET into routine care plans for patients with T2DM and CAN, offering a holistic approach to disease management. Moreover, the inclusion of CET can complement existing pharmacological treatments, potentially enhancing overall treatment efficacy and patient outcomes. These findings support the integration of CET into standard clinical practice for managing T2DM with CAN, promoting a multidisciplinary approach to patient care.

## 5. Strength

The study demonstrates significant strengths in its methodology and findings. One of the primary strengths is the rigorous design, employing an RCT with an intention-to-treat approach, which enhances the reliability and validity of the results. The sample size calculation, based on previous studies, enhances the study’s power to detect significant differences. The comprehensive pre-training assessments, including blood glucose monitoring, blood pressure, and heart rate assessments, ensured participant safety and accurate baseline data. The use of established and validated measurement tools, such as the ADDQoL-19, provided robust assessments of QoL. Additionally, the training program was meticulously designed to align with joint guidelines from the ACSM and the ADA, ensuring the intervention was both safe and effective.

## 6. Limitations

Despite its strengths, the study has several limitations that should be considered. One significant limitation is the lack of blinding for participants, assessors, and intervention providers. While participants were aware of their group allocation due to the nature of the exercise intervention, the lack of blinding could introduce bias. The relatively short duration of the intervention (13 weeks) may not capture the long-term effects of CET on the studied health parameters. Furthermore, the study’s exclusion criteria, which omitted individuals with severe comorbidities or those unable to participate in the exercises, limit the generalizability of the findings to all individuals with T2DM and CAN. Another limitation is the generalizability to older patients. Although the study eligibility criteria included participants up to 70 years old, the findings revealed that the participants were all under 60 years old. This is due to our inclusion criteria based on the Ewing battery, which only considered early and definite CAN subjects, excluding patients with advanced or severe CAN, which is more common in older T2DM patients. Additionally, the demographics of our participants, primarily working professionals who are not yet of retirement age, also contributed to this age distribution. The generalizability of the findings is also limited by the specific population recruited from certain centers, which may not represent broader demographics or settings. Potential confounding factors, such as variations in diet, lifestyle changes outside the intervention, and adherence to the exercise program, could have influenced the results despite efforts to control these variables. We acknowledge that no detailed data analysis was performed on the nutritional/diet status of the participants, which is a limitation of the study. Additionally, our study did not record the specific types of oral diabetes medications in detail, categorizing them broadly as oral antidiabetic drugs. This lack of specificity in medication history is a limitation of the study and should be addressed in future research to better understand the potential interactions between different pharmacological treatments and exercise interventions. Lastly, the control group received usual care without additional interventions, potentially overlooking placebo effects or the benefits of increased attention and monitoring in the intervention group.

## 7. Future Perspectives

Future research should focus on extending the follow-up periods to assess the sustained benefits and long-term impacts of CET on individuals with T2DM and CAN. Studies could also explore the impact of CET on a broader range of T2DM patients, including those with more advanced stages of CAN, to enhance the generalizability of the findings. Incorporating double-blind designs, where both participants and assessors are blinded, could further minimize bias and improve the reliability of the results. Investigating the underlying mechanisms through which CET influences various health parameters at the molecular and cellular levels could provide deeper insights into the physiological adaptations induced by the intervention. Comparative studies that evaluate CET against other types of interventions, such as pharmacological treatments or alternative exercise modalities, would help determine the relative efficacy and potential synergistic effects of combined therapies. Implementing double-blind designs where feasible or using objective measures and automated assessments could further reduce bias and enhance the validity of future findings.

## 8. Conclusions

This study provides compelling evidence that CET significantly improves cardiac autonomic modulation, metabolic profile, body composition, cardiorespiratory fitness, and QoL in individuals with T2DM and CAN. The findings highlight the multifaceted benefits of combining aerobic and resistance exercises, suggesting that CET should be integrated into the standard management strategies for T2DM patients. While the study has certain limitations, its strengths in design and comprehensive approach offer valuable insights into the therapeutic potential of CET. Future research should build on these findings to explore long-term effects and broader applicability, ultimately aiming to optimize exercise interventions for improved health outcomes in the diabetic population.

## Figures and Tables

**Table 1:** Comparison of demographics and subject characteristics at baseline.

Variables		CET (*n* = 48)	Control (*n* = 48)	*p*-Value
		Mean ± SD	Mean ± SD	
Age (years)		46.38 ± 7.55	47.56 ± 7.88	0.45
Sex	Male/Female	29/19	33/15	0.52
Marital status	Married/Single/Widower/Divorced	40/4/2/2	45/2/1/0	0.34
Professional activity	Working/Not working	40/8	33/15	0.15
Education	Primary and vocational/Preuniversity/Higher	8/19/10	11/30/18	0.07
Place of residence	Rural/Urban	7/41	4/44	0.33
Smoking	Never/Past/Present	40/8/0	40/4/4	0.06
Alcohol	Drinking/Not drinking	0/48	2/46	0.49
Duration of diabetes		7.14 ± 4.84	7.04 ± 4.92	0.91
Drugs	Oral antidiabetic/Insulin/Diet	45/2/1	43/2/3	0.59
***CARTs***				
**Diagnosis, *n* (*n*%)**				
Early		21 (43.8)	19 (39.6)	0.83
Definite		27 (56.3)	29 (60.4)	0.83
E:I ratio		0.93 ± 0.12	0.86 ± 0.23	0.80
ΔHR		8.51 ± 3.73	7.56 ± 3.78	0.22
VR		1.06 ± 0.26	1.13 ± 0.18	0.15
ΔDBP (mmHg)		4.06 ± 2.29	4.21 ± 1.66	0.72
30:15 ratio		0.95 ± 0.09	0.97 ± 0.07	0.33
ΔSBP (mmHg)		5.69 ± 2.57	6.02 ± 2.23	0.49

CARTs—cardiovascular autonomic reflex tests; E:I ratio—ratio of the average of longest R-R interval during expiration and the shortest R-R interval during inspiration of the deep breathing test; 30:15 ratio—ratio of the longest R-R interval during 30 s and the shortest R-R interval during 15th s of the head-up tilt test; VR—valsalva ratio; ΔHR—change in R-R intervals during six consecutive cycles of deep inspiration and expiration; ΔSBP—change in systolic blood pressure during head-up tilt test; ΔDBP—change in diastolic blood pressure during hand grip t Primary and Secondary Outcomes at Baseline.

**Table 2:** Comparison of primary and secondary outcome measures between the groups at baseline.

Variables	CET (*n* = 48)	Control (*n* = 48)	*p*-Value
	Mean ± SD	Mean ± SD	
**Resting HRV parameters**			
SDNN (ms)	33.55 ± 4.93	33.75 ± 4.23	0.87
RMSSD (ms)	28.97 ± 5.99	31.28 ± 5.73	0.05
pNN50 (%)	1.76 ± 0.25	1.83 ± 0.27	0.24
TP (ms^2^)	2260.52 ± 1585.84	1952.51 ± 1384.43	0.31
LF power (ms^2^)	279.12 ± 44.64	279.05 ± 50.87	0.99
HF power (ms^2^)	295.67 ± 42.89	296.89 ± 51.09	0.89
LF/HF ratio	1.34 ± 0.45	1.37 ± 0.48	0.70
**Heart rate recovery**			
Peak HR (bpm)	167.56 ± 10.33	169.56 ± 12.21	0.38
HRR_30s_ (bpm)	3.18 ± 1.57	2.89 ± 1.37	0.33
HRR_1_ (bpm)	18.12 ± 3.80	18.66 ± 4.02	0.50
HRR_2_ (bpm)	34.56 ± 6.50	33.64 ± 4.55	0.42
HRR_3_ (bpm)	60.41 ± 6.88	59.43 ± 6.69	0.48
**Cardiovascular function**			
HR_rest_ (bpm)	77.15 ± 7.57	76.35 ± 6.94	0.60
SBP (mmHg)	129.44 ± 16.28	130.83 ± 18.18	0.69
DBP (mmHg)	86.33 ± 5.43	86.33 ± 7.33	0.70
**Glycemic control**			
FBG (mg/dL)	133.8 ± 28.73	141.67 ± 29.20	0.18
PPBG (mg/dL)	147.44 ± 29.22	173.50 ± 42.33	0.50
HbA1c (%)	6.83 ± 0.23	6.89 ± 0.15	0.11
**Body composition**			
BMI (kg/m^2^)	27.15 ± 2.96	26.69 ± 2.72	0.43
WC (cm)	96.79 ± 2.30	96.73 ± 2.21	0.89
HC (cm)	103.29 ± 2.75	103.42 ± 2.59	0.81
WHR	0.93 ± 0.31	0.92 ± 0.04	0.23
PBF	34.27 ± 0.56	34.38 ± 0.47	0.31
**Lipid profile**			
TC (mg/dL)	212.19 ± 14.53	214.77 ± 4.16	0.38
TG (mg/dL)	166.88 ± 9.60	170.42 ± 7.77	0.05
LDL-C (mg/dL)	133.13 ± 10.75	136.56 ± 11.25	0.12
HDL-C (mg/dL)	51.65 ± 3.17	52.58 ± 2.80	0.12
**Cardiorespiratory fitness**			
VO_2max_ (mL/kg/min)	25.69 ± 5.59	24.73 ± 4.83	0.37
**Diabetes Dependent QoL (ADDQoL-19)**			
ADDQoL-19-GQoL	1.23 ± 2.75	1.19 ± 1.73	0.90
ADDQoL-19-DDQoL	−1.42 ± 0.67	−1.37 ± 0.67	0.76
ADDQoL-19-AWIS	−0.48 ± 2.63	−0.60 ± 2.82	0.82

SDNN-standard deviation of N-N intervals; RMSSD-root mean square of successive differences between adjacent R-R intervals; pNN50-Proportion of differences in consecutive N-N intervals that are longer than 50 ms; TP-total power; LF-low frequency; HF-high frequency; LF/HF ratio-ratio of low- and high-frequency power; HRpeak-peak heart rate; HRR-heart rate recovery; BMI-body mass index; HR_rest_-resting heart rate; SBP-systolic blood pressure; DBP-diastolic blood pressure; FBG-fasting blood glucose; PPBG-post-prandial blood glucose; HbA1c-glycosylated hemoglobin; VO_2max_-volume of maximum oxygen consumption; Waist circumference; HC-Hips circumference; WHR-Waist–hip ratio; PBF-percentage body fat; TC-total cholesterol; TG-triglycerides; LDL-C-low-density lipoprotein cholesterol; HDL-C-high-density lipoprotein cholesterol; ADDQoL-19-19-item Audit of Diabetes Dependent Quality of Life; QoL-quality of life; GQoL-general quality of life, DDQoL-diabetes-dependent quality of life, AWIS-average weighted impact score.

**Table 3:** Results of the 2 × 2 mixed ANOVA for the between and within-group comparison.

Variables	CET (*n* = 48) (End of 13th Week)	Control (*n* = 48) (End of 13th Week)	Source of Variation	*p*-Value	F-Value	pη^2^
**Heart rate variability**						
SDNN (ms)	47.47 ± 8.87	33.65 ± 4.26	Time	<0.01 **	127.40	0.57
			Group × Time	<0.01 **	131.43	0.58
			Group	<0.01 **	43.12	0.31
RMSSD (ms)	51.61 ± 8.22	31.10 ± 5.93	Time	<0.01 **	196.23	0.67
			Group × Time	<0.01 **	202.62	0.68
			Group	<0.01 **	72.05	0.43
pNN50 (%)	2.68 ± 0.75	1.82 ± 0.33	Time	<0.01 **	78.69	0.45
			Group × Time	<0.01 **	82.35	0.46
			Group	<0.01 **	27.15	0.22
TP (ms^2^)	4084.72 ± 1740.65	1952.41 ± 1385.02	Time	<0.01 **	41.61	0.30
			Group × Time	<0.01 **	41.62	0.30
			Group	<0.01 **	19.15	0.16
LF power (ms^2^)	166.97 ± 42.45	269.01 ± 55.56	Time	<0.01 **	78.51	0.45
			Group × Time	<0.01 **	54.83	0.36
			Group	<0.01 **	50.85	0.35
HF power (ms^2^)	520.85 ± 103.46	305.54 ± 101.93	Time	<0.01 **	132.09	0.58
			Group × Time	<0.01 **	113.28	0.54
			Group	<0.01 **	70.49	0.42
LF/HF ratio	0.54 ± 0.21	1.34 ± 0.49	Time	<0.01 **	190.84	0.67
			Group × Time	<0.01 **	156.90	0.62
			Group	<0.01 **	26.03	0.21
**Heart rate recovery**						
Peak HR (bpm)	134.93 ± 10.07	167.70 ± 12.38	Time	<0.01 **	226.15	0.70
			Group × Time	<0.01 **	229.92	0.71
			Group	<0.01 **	15.05	0.38
HRR_30s_ (bpm)	6.43 ± 3.03	3.89 ± 4.30	Time	<0.01 **	32.01	0.24
			Group × Time	<0.01 **	8.97	0.08
			Group	<0.01 **	10.36	0.09
HRR_1_ (bpm)	34.06 ± 7.77	18.87 ± 4.06	Time	<0.01 **	155.63	0.62
			Group × Time	<0.01 **	147.70	0.61
			Group	<0.01 **	76.21	0.44
HRR_2_ (bpm)	65.81 ± 11.64	32.72 ± 4.36	Time	<0.01 **	224.68	0.70
			Group × Time	<0.01 **	252.66	0.72
			Group	<0.01 **	232.42	0.71
HRR_3_ (bpm)	76.41 ± 7.55	58.31 ± 6.78	Time	<0.01 **	73.56	0.43
			Group × Time	<0.01 **	97.50	0.50
			Group	0.57	0.31	0.00
**Cardiovascular function**						
HR_rest_	77.15 ± 7.57	76.35 ± 6.94	Time	<0.01 **	17.74	0.15
			Group × Time	<0.01 **	12.82	0.12
			Group	0.06	3.46	0.03
SBP (mmHg)	129.44 ± 16.28	130.83 ± 18.18	Time	<0.01 **	16.44	0.14
			Group × Time	<0.01 **	12.49	0.11
			Group	0.13	2.27	0.02
DBP (mmHg)	86.33 ± 5.43	86.33 ± 7.33	Time	<0.01 **	19.22	0.17
			Group × Time	0.85	0.03	0.00
			Group	0.57	0.31	0.00
**Glycemic control**						
FBG (mg/dL)	110.48 ± 10.11	141.60 ± 28.63	Time	<0.01 **	25.89	0.21
			Group × Time	<0.01 **	25.61	0.21
			Group	<0.01 **	17.40	0.15
PPBG (mg/dL)	147.44 ± 25.72	174.38 ± 43.32	Time	<0.01 **	46.27	0.33
			Group × Time	<0.01 **	54.63	0.36
			Group	0.29	4.90	0.05
HbA1c (%)	6.25 ± 0.40	6.74 ± 0.49	Time	<0.01 **	49.76	0.34
			Group × Time	<0.01 **	17.30	0.15
			Group	<0.01 **	30.54	0.24
**Body composition**						
BMI (kg/m^2^)	27.53 ± 2.88	26.69 ± 2.72	Time	<0.01 **	0.48	0.00
			Group × Time	<0.01 **	0.48	0.00
			Group	0.20	1.62	0.01
WC (cm)	90.71 ± 4.53	96.60 ± 2.56	Time	<0.01 **	68.41	0.42
			Group × Time	<0.01 **	63.01	0.40
			Group	<0.01 **	34.32	0.26
HC (cm)	96.00 ± 7.11	102.42 ± 3.54	Time	<0.01 **	51.92	0.35
			Group × Time	<0.01 **	29.53	0.23
			Group	<0.01 **	22.64	0.19
WHR	0.93 ± 0.03	0.93 ± 0.02	Time	<0.01 **	2.64	0.02
			Group × Time	<0.01 **	3.63	0.03
			Group	0.75	0.09	0.001
PBF	28.34 ± 3.90	34.00 ± 1.30	Time	<0.01 **	113.91	0.54
			Group × Time	<0.01 **	88.18	0.48
			Group	<0.01 **	87.45	0.48
**Lipid profile**						
TC (mg/dL)	155.90 ± 32.93	211.40 ± 21.70	Time	<0.01 **	114.19	0.54
			Group × Time	<0.01 **	89.81	0.48
			Group	<0.01 **	65.62	0.41
TG (mg/dL)	124.85 ± 19.03	169.42 ± 9.74	Time	<0.01 **	188.61	0.66
			Group × Time	<0.01 **	171.48	0.64
			Group	<0.01 **	148.32	0.61
LDL-C (mg/dL)	119.96 ± 17.88	133.63 ± 13.35	Time	<0.01 **	29.37	0.23
			Group × Time	<0.01 **	11.85	0.11
			Group	<0.01 **	13.28	0.12
HDL-C (mg/dL)	63.46 ± 7.78	54.15 ± 5.87	Time	<0.01 **	96.98	0.50
			Group × Time	<0.01 **	56.96	0.37
			Group	<0.01 **	24.43	0.20
**Cardiorespiratory fitness**						
VO_2max_ (mL/kg/min)	40.04 ± 10.27	24.01 ± 3.76	Time	<0.01 **	69.43	0.42
			Group × Time	<0.01 **	84.80	0.47
			Group	<0.01 **	62.78	0.40
**Diabetes Dependent QoL (ADDQoL-19)**						
ADDQoL-19-GQoL	2.25 ± 0.75	1.19 ± 1.84	Time	<0.01 **	15.78	0.14
			Group × Time	<0.01 **	15.78	0.14
			Group	<0.01 **	3.45	0.03
ADDQoL-19-DDQoL	0.58 ± 0.49	−1.50 ± 0.74	Time	<0.01 **	225.00	0.70
			Group × Time	<0.01 **	289.00	0.75
			Group	<0.01 **	74.68	0.44
ADDQoL-19-AWIS	1.98 ± 1.15	−0.25 ± 2.32	Time	<0.01 **	18.72	0.16
			Group × Time	<0.01 **	10.48	0.10
			Group	<0.01 **	11.51	0.10

SDNN—standard deviation of N-N intervals; RMSSD—root mean square of successive differences between adjacent R-R intervals; pNN50—Proportion of differences in consecutive N-N intervals that are longer than 50 ms; TP—total power; LF—low frequency; HF—high frequency; LF/HF ratio—ratio of low- and high-frequency power; HRpeak—peak heart rate; HRR—heart rate recovery; BMI—body mass index; HR_rest_—resting heart rate; SBP—systolic blood pressure; DBP—diastolic blood pressure; FBG—fasting blood glucose; PPBG—post-prandial blood glucose; HbA1c—glycosylated hemoglobin; VO_2max_—volume of maximum oxygen consumption; Waist circumference; HC—Hips circumference; WHR—Waist–hip ratio; PBF—percentage body fat; TC—total cholesterol; TG—triglycerides; LDL-C—low-density lipoprotein cholesterol; HDL-C—high-density lipoprotein cholesterol; ADDQoL-19—19-item Audit of Diabetes Dependent Quality of Life; QoL—quality of life; GQoL—general quality of life, DDQoL—diabetes-dependent quality of life, AWIS—average weighted impact score; pη^2^—partial eta square; **: Significance level of *p* < 0.01.

## Data Availability

The datasets generated and analyzed during the current study are available from the corresponding author upon reasonable request. For any inquiries regarding this paper, please contact Saurabh Sharma (ssharma@jmi.ac.in).

## References

[1] Galicia-Garcia U., Benito-Vicente A., Jebari S., Larrea-Sebal A., Siddiqi H., Uribe K.B., Ostolaza H., Martín C. (2020). Pathophysiology of Type 2 Diabetes Mellitus. Int. J. Mol. Sci..

[2] Duque A., Mediano M.F.F., De Lorenzo A., Rodrigues L.F. (2021). Cardiovascular autonomic neuropathy in diabetes: Pathophysiology, clinical assessment and implications. World J. Diabetes.

[3] Colberg S.R., Sigal R.J., Fernhall B., Regensteiner J.G., Blissmer B.J., Rubin R.R., Chasan-Taber L., Albright A.L., Braun B. (2010). Exercise and type 2 diabetes: The American College of Sports Medicine and the American Diabetes Association: Joint position statement. Diabetes Care.

[4] Ambelu T., Teferi G. (2023). The impact of exercise modalities on blood glucose, blood pressure and body composition in patients with type 2 diabetes mellitus. BMC Sports Sci. Med. Rehabil..

[5] Syeda U.S.A., Battillo D., Visaria A., Malin S.K. (2023). The importance of exercise for glycemic control in type 2 diabetes. Am. J. Med. Open.

[6] Kirwan J.P., Sacks J., Nieuwoudt S. (2017). The essential role of exercise in the management of type 2 diabetes. Cleve Clin. J. Med..

[7] Richter E.A., Hargreaves M. (2013). Exercise, GLUT4, and Skeletal Muscle Glucose Uptake. Physiol. Rev..

[8] Merz K.E., Thurmond D.C. (2020). Role of Skeletal Muscle in Insulin Resistance and Glucose Uptake. Compr. Physiol..

[9] Currier B.S., McLeod J.C., Banfield L., Beyene J., Welton N.J., D’Souza A.C., Keogh J.A.J., Lin L., Coletta G., Yang A. (2023). Resistance training prescription for muscle strength and hypertrophy in healthy adults: A systematic review and Bayesian network meta-analysis. Br. J. Sports Med..

[10] Kelley G.A., Kelley K.S., Stauffer B.L. (2023). Effects of resistance training on body weight and body composition in older adults: An inter-individual response difference meta-analysis of randomized controlled trials. Sci. Prog..

[11] Coffey V.G., Hawley J.A. (2017). Concurrent exercise training: Do opposites distract?. J. Physiol..

[12] Zaki S., Sharma S., Vats H. (2023). Effectiveness of concurrent exercise training in people with type 2 diabetes: A systematic review and meta-analysis. Physiother. Theory Pract..

[13] Gordan R., Gwathmey J.K., Xie L.H. (2015). Autonomic and endocrine control of cardiovascular function. World J. Cardiol..

[14] Leon B.M., Maddox T.M. (2015). Diabetes and cardiovascular disease: Epidemiology, biological mechanisms, treatment recommendations and future research. World J. Diabetes.

[15] Zaki S., Alam F., Faizan M., Sharma S., Naqvi I.H. (2024). Association between heart rate variability and cardiorespiratory fitness in individuals with type 2 diabetes mellitus: A cross-sectional study. J. Human. Sport. Exerc..

[16] Kaze A.D., Agoons D.D., Santhanam P., Erqou S., Ahima R.S., Echouffo-Tcheugui J.B. (2022). Correlates of cardiorespiratory fitness among overweight or obese individuals with type 2 diabetes. BMJ Open Diabetes Res. Care.

[17] Bouamra M., Zouhal H., Ratel S., Makhlouf I., Bezrati I., Chtara M., Behm D.G., Granacher U., Chaouachi A. (2022). Concurrent Training Promotes Greater Gains on Body Composition and Components of Physical Fitness Than Single-Mode Training (Endurance or Resistance) in Youth With Obesity. Front. Physiol..

[18] Wang S., Ji X., Zhang Z., Xue F. (2020). Relationship between Lipid Profiles and Glycemic Control Among Patients with Type 2 Diabetes in Qingdao, China. Int. J. Environ. Res. Public Health.

[19] Sabag A., Chang C.R., Francois M.E., Keating S.E., Coombes J.S., Johnson N.A., Pastor-Valero M., Rey Lopez J.P. (2023). The Effect of Exercise on Quality of Life in Type 2 Diabetes: A Systematic Review and Meta-analysis. Med. Sci. Sports Exerc..

[20] Silva L.R., Gentil P., Seguro C.S., de Oliveira J.C., Silva M.S., Marques V.A., Beltrame T., Rebelo A.C. (2022). High-Intensity Interval Training Improves Cardiac Autonomic Function in Patients with Type 2 Diabetes: A Randomized Controlled Trial. Biology.

[21] Hamasaki H. (2023). The Effect of Exercise on Cardiovascular Autonomic Nervous Function in Patients with Diabetes: A Systematic Review. Healthcare.

[22] General Assembly of the World Medical Association (2014). World Medical Association Declaration of Helsinki: Ethical principles for medical research involving human subjects. J. Am. Coll. Dent..

[23] American Diabetes Association (2011). Diagnosis and classification of diabetes mellitus. Diabetes Care.

[24] Ewing D.J., Martyn C.N., Young R.J., Clarke B.F. (1985). The value of cardiovascular autonomic function tests: 10 years experience in diabetes. Diabetes Care.

[25] (2013). ACSM’s Guidelines for Exercise Testing and Prescription.

[26] Bassi D., Mendes R.G., Arakelian V.M., Caruso F.C.R., Cabiddu R., Júnior J.C.B., Arena R., Borghi-Silva A. (2016). Potential Effects on Cardiorespiratory and Metabolic Status After a Concurrent Strength and Endurance Training Program in Diabetes Patients—A Randomized Controlled Trial. Sports Med. Open.

[27] Catai A.M., Pastre C.M., Godoy M.F., Silva E.D., Takahashi A.C.M., Vanderlei L.C.M. (2020). Heart rate variability: Are you using it properly? Standardisation checklist of procedures. Braz. J. Phys. Ther..

[28] Bhati P., Hussain M.E. (2019). Sleep duration is a significant predictor of cardiac autonomic neuropathy in type 2 diabetes mellitus. Prim. Care Diabetes.

[29] (1996). Heart rate variability. Standards of measurement, physiological interpretation, and clinical use. Task Force of the European Society of Cardiology and the North American Society of Pacing and Electrophysiology. Eur. Heart J..

[30] Peltenburg P.J., Kallas D., Bos J.M., Lieve K.V., Franciosi S., Roston T.M., Denjoy I., Sorensen K.B., Ohno S., Roses-Noguer F. (2022). An international multicenter cohort study on β-blockers for the treatment of symptomatic children with catecholaminergic polymorphic ventricular tachycardia. Circulation.

[31] Bhati P., Hussain M.E. (2021). Leisure-Time Physical Activity and Glycemic Control Independently Predicts Cardiac Autonomic Neuropathy in Type 2 Diabetes Mellitus. J. Phys. Act. Health.

[32] Okutucu S., Karakulak U.N., Aytemir K., Oto A. (2011). Heart rate recovery: A practical clinical indicator of abnormal cardiac autonomic function. Expert. Rev. Cardiovasc. Ther..

[33] Gibbons R.J., Balady G.J., Beasley J.W., Bricker J.T., Duvernoy W.F., Froelicher V.F., Mark D.B., Marwick T.H., McCallister B.D., Thompson P.D. (1997). ACC/AHA Guidelines for Exercise Testing. A report of the American College of Cardiology/American Heart Association Task Force on Practice Guidelines (Committee on Exercise Testing). J. Am. Coll. Cardiol..

[34] Shetler K., Marcus R., Froelicher V.F., Vora S., Kalisetti D., Prakash M., Do D., Myers J. (2001). Heart rate recovery: Validation and methodologic issues. J. Am. Coll. Cardiol..

[35] Muntner P., Shimbo D., Carey R.M., Charleston J.B., Gaillard T., Misra S., Myers M.G., Ogedegbe G., Schwartz J.E., Townsend R.R. (2019). Measurement of blood pressure in humans: A scientific statement from the American Heart Association. Hypertension.

[36] Ogedegbe G., Pickering T. (2010). Principles and techniques of blood pressure measurement. Cardiol. Clin..

[37] Nuttall F.Q. (2015). Body Mass Index: Obesity, BMI, and Health: A Critical Review. Nutr. Today.

[38] Ma W.Y., Yang C.Y., Shih S.R., Hsieh H.J., Hung C.S., Chiu F.C., Lin M.S., Liu P.H., Hua C.H., Hsein Y.C. (2013). Measurement of Waist Circumference: Midabdominal or iliac crest?. Diabetes Care.

[39] Katz E.G., Stevens J., Truesdale K.P., Cai J., Adair L.S., North K.E. (2011). Hip circumference and incident metabolic risk factors in Chinese men and women: The People’s Republic of China study. Metab. Syndr. Relat. Disord..

[40] Jackson A.S., Pollock M.L., Ward A. (1980). Generalized equations for predicting body density of women. Med. Sci. Sports Exerc..

[41] Siri W. (1961). Body Composition from Fluid Spaces and Density: Analysis of Methods.

[42] Bradley C., Todd C., Gorton T., Symonds E., Martin A., Plowright R. (1999). The development of an individualized questionnaire measure of perceived impact of diabetes on quality of life: The ADDQoL. Qual. Life Res..

[43] Bradley C. (2017). The Audit of Diabetes-Dependent Quality of Life (ADDQoL). User Guidelines. https://healthpsychologyresearch.com/.

[44] Beltz N.M., Gibson A.L., Janot J.M., Kravitz L., Mermier C.M., Dalleck L.C. (2016). Graded Exercise Testing Protocols for the Determination of VO(2)max: Historical Perspectives, Progress, and Future Considerations. J. Sports Med..

[45] Trabulo M., Mendes M., Mesquita A., Seabra-Gomes R. (1994). [Does the modified Bruce protocol induce physiological stress equal to that of the Bruce protocol?]. Rev. Port. Cardiol..

[46] Colberg S.R. (2013). Exercise and Diabetes: A Clinician’s Guide to Prescribing Physical Activity.

[47] Colberg S.R. (2017). Key Points from the Updated Guidelines on Exercise and Diabetes. Front. Endocrinol..

[48] Jorge M.L.M.P., de Oliveira V.N., Resende N.M., Paraiso L.F., Calixto A., Diniz A.L.D., Resende E.S., Ropelle E.R., Carvalheira J.B., Espindola F.S. (2011). The effects of aerobic, resistance, and combined exercise on metabolic control, inflammatory markers, adipocytokines, and muscle insulin signaling in patients with type 2 diabetes mellitus. Metabolism.

[49] American Diabetes Association Professional Practice Committee (2021). 2. Classification and Diagnosis of Diabetes: Standards of Medical Care in Diabetes—2022. Diabetes Care.

[50] Karvonen M.J., Kentala E., Mustala O. (1957). The effects of training on heart rate; a longitudinal study. Ann. Med. Exp. Biol. Fenn..

[51] Annibalini G., Lucertini F., Agostini D., Vallorani L., Gioacchini A., Barbieri E., Guescini M., Casadei L., Passalia A., Del Sal M. (2017). Concurrent Aerobic and Resistance Training Has Anti-Inflammatory Effects and Increases Both Plasma and Leukocyte Levels of IGF-1 in Late Middle-Aged Type 2 Diabetic Patients. Oxidative Med. Cell. Longev..

[52] Saeidi A., Soltani M., Daraei A., Nohbaradar H., Haghighi M.M., Khosravi N., Johnson K.E., Laher I., Hackney A.C., VanDusseldorp T.A. (2021). The Effects of Aerobic-Resistance Training and Broccoli Supplementation on Plasma Dectin-1 and Insulin Resistance in Males with Type 2 Diabetes. Nutrients.

[53] Brzycki M. (1993). Strength Testing—Predicting a One-Rep Max from Reps-to-Fatigue. J. Phys. Educ. Recreat. Danc..

[54] American Diabetes Association (2022). Standards of Care in Diabetes—2023 Abridged for Primary Care Providers. Clin. Diabetes.

[55] Gupta S.K. (2011). Intention-to-treat concept: A review. Perspect. Clin. Res..

[56] Su X., He J., Cui J., Li H., Men J. (2022). The effects of aerobic exercise combined with resistance training on inflammatory factors and heart rate variability in middle-aged and elderly women with type 2 diabetes mellitus. Ann. Noninvasive Electrocardiol..

[57] Routledge F.S., Campbell T.S., McFetridge-Durdle J.A., Bacon S.L. (2010). Improvements in heart rate variability with exercise therapy. Can. J. Cardiol..

[58] Picard M., Tauveron I., Magdasy S., Benichou T., Bagheri R., Ugbolue U.C., Navel V., Dutheil F. (2021). Effect of exercise training on heart rate variability in type 2 diabetes mellitus patients: A systematic review and meta-analysis. PLoS ONE.

[59] Vinik A.I., Casellini C., Parson H.K., Colberg S.R., Nevoret M.-L. (2018). Cardiac Autonomic Neuropathy in Diabetes: A Predictor of Cardiometabolic Events. Front. Neurosci..

[60] Nascimentoa P.M., Vieiraa M.C., Sperandeib S., Serraa S.M. (2016). Supervised exercise improves autonomic modulation in participants in cardiac rehabilitation programs. Rev. Port. Cardiol..

[61] Dewar A., Kass L., Stephens R.C.M., Tetlow N., Desai T. (2023). Heart Rate Recovery Assessed by Cardiopulmonary Exercise Testing in Patients with Cardiovascular Disease: Relationship with Prognosis. Int. J. Environ. Res. Public Health.

[62] Romero S.A., Minson C.T., Halliwill J.R. (2017). The cardiovascular system after exercise. J. Appl. Physiol..

[63] Zaki S., Moiz J.A., Bhati P., Menon G.R. (2022). Efficacy of high-intensity interval training on cardiac autonomic modulation in cardiovascular diseases and lifestyle disorders: A systematic review and meta-analysis. Comp. Exerc. Physiol..

[64] Abulmeaty M.M.A., Aljuraiban G.S., Alaidarous T.A., Alkahtani N.M. (2020). Body Composition and the Components of Metabolic Syndrome in Type 2 Diabetes: The Roles of Disease Duration and Glycemic Control. Diabetes Metab. Syndr. Obes..

[65] Pandey A., Swift D.L., McGuire D.K., Ayers C.R., Neeland I.J., Blair S.N., Johannsen N., Earnest C.P., Berry J.D., Church T.S. (2015). Metabolic Effects of Exercise Training Among Fitness-Nonresponsive Patients With Type 2 Diabetes: The HART-D Study. Diabetes Care.

[66] Olson K.L., Neiberg R.H., Espeland M.A., Johnson K.C., Knowler W.C., Pi-Sunyer X., Staiano A.E., Wagenknecht L.E., Wing R.R. (2020). Waist Circumference Change During Intensive Lifestyle Intervention and Cardiovascular Morbidity and Mortality in the Look AHEAD Trial. Obesity.

[67] Zhao X., He Q., Zeng Y., Cheng L. (2021). Effectiveness of combined exercise in people with type 2 diabetes and concurrent overweight/obesity: A systematic review and meta-analysis. BMJ Open.

[68] Warren J.L., Hunter G.R., Gower B.A., Bamman M.M., Windham S.T., Moellering D.R., Fisher G. (2020). Exercise Effects on Mitochondrial Function and Lipid Metabolism during Energy Balance. Med. Sci. Sports Exerc..

[69] Pesta D.H., Goncalves R.L.S., Madiraju A.K., Strasser B., Sparks L.M. (2017). Resistance training to improve type 2 diabetes: Working toward a prescription for the future. Nutr. Metab..

[70] Nystoriak M.A., Bhatnagar A. (2018). Cardiovascular Effects and Benefits of Exercise. Front. Cardiovasc. Med..

[71] Hughes D.C., Ellefsen S., Baar K. (2018). Adaptations to Endurance and Strength Training. Cold Spring Harb. Perspect. Med..

[72] Ren J., Xiao H. (2023). Exercise for Mental Well-Being: Exploring Neurobiological Advances and Intervention Effects in Depression. Life.

[73] Gacesa J.Z., Klasnja A.V., Grujic N.G. (2013). Changes in strength, endurance, and fatigue during a resistance-training program for the triceps brachii muscle. J. Athl. Train..

